# Quantitative Scintigraphy Imaging of Lingual Raynaud’s Phenomenon Using 3-Dimensional-Ring Cadmium-Zinc-Telluride Single-Photon Emission Computed Tomography/Computed Tomography

**DOI:** 10.3390/tomography8040171

**Published:** 2022-08-17

**Authors:** Ik Dong Yoo, In Young Jo, Geum Cheol Jeong, Yong Kyun Won, Du Shin Jeong, Sang Mi Lee

**Affiliations:** 1 Department of Nuclear Medicine, Soonchunhyang University Cheonan Hospital, 31 Suncheonhyang 6-gil, Dongnam-gu, Cheonan 31151, Korea; 2 Department of Radiation Oncology, Soonchunhyang University Cheonan Hospital, 31 Suncheonhyang 6-gil, Dongnam-gu, Cheonan 31151, Korea; 3 Department of Neurology, Soonchunhyang University Cheonan Hospital, 31 Suncheonhyang 6-gil, Dongnam-gu, Cheonan 31151, Korea

**Keywords:** cadmium-zinc-telluride, Raynaud’s phenomenon, scintigraphy, single-photon emission computed tomography/computed tomography

## Abstract

Perfusion scintigraphy with the acquisition of planar blood flow and pool images of bilateral hands has been used to aid diagnosis and to evaluate treatment response to Raynaud’s phenomenon (decreased blood flow to hand or foot). However, because of the difficulty in imaging the tongue area with a conventional gamma camera, perfusion scintigraphy imaging of patients with lingual Raynaud’s phenomenon has yet to be reported. Here, we report the case of a 59-year-old man with lingual Raynaud’s phenomenon in which blood pool imaging of the tongue was performed using three-dimensional (3D)-ring cadmium-zinc-telluride (CZT) single-photon emission computed tomography/computed tomography (SPECT/CT). During follow-up, the patient’s lingual symptoms had worsened, and follow-up blood pool SPECT/CT images also revealed decreased blood pool uptake of the tongue, showing a decreased blood pool of more than 25% on quantitative analysis. This case suggests that blood pool imaging of the tongue using 3D-ring CZT SPECT/CT has clinical significance in evaluating patients with lingual Raynaud’s phenomenon.

## 1. Introduction

Raynaud’s phenomenon is caused by the deregulated vasoconstriction of peripheral arteries and arterioles and typically manifests as intermittent episodes of skin color change (pallor, cyanosis, and rubor), swelling, and paresthesia [1,2]. The overall prevalence of Raynaud’s phenomenon is between 1.6% and 7.2% in the general population, with a higher prevalence in the people who reside in colder climates [1,3]. Raynaud’s phenomenon is triggered by cold exposure, vibration, trauma, or emotional stress and is classified into primary form (Raynaud’s disease) and secondary form (Raynaud syndrome), which is related to rheumatic disease, arterial occlusive disease, neurological disease, and medications [1,2]. This phenomenon usually affects the fingers and/or toes but can also affect other areas of the body such as the nose, ears, and tongue [1,4].

Although the diagnosis of Raynaud’s phenomenon is mainly based on the history and physical examination of patients, several studies have shown that perfusion scintigraphy can aid in its diagnosis [5,6]. Radiopharmaceuticals, such as Tc-99m red blood cells, Tc-99m pertechnetate, and Tc-99m methylene diphosphonate (MDP), are used in perfusion scintigraphy for diagnosing Raynaud’s phenomenon [5]. After the intravenous injection of radiopharmaceuticals, planar blood flow and blood pool images of the bilateral hands were acquired with or without cold stimulation, and the perfusion status of the fingers was evaluated by measuring the uptake by the fingers [5,6]. Recently, with the advancement of medical imaging technologies, three-dimensional (3D)-ring cadmium-zinc-telluride (CZT) single-photon emission tomography/computed tomography (SPECT/CT) has been newly introduced [7], and 3D-ring CZT SPECT/CT allows rapid 3D imaging acquisition with superior image sensitivity and quality compared to that of the planar gamma scintigraphy and conventional SPECT and offers reliable quantification of uptake [8,9]. Therefore, this SPECT/CT system is considered to provide positron emission tomography-like utilization in clinical settings [9].

Here, we report a case of lingual Raynaud’s phenomenon who showed decreased blood pool uptake in the tongue on 3D-ring CZT SPECT/CT images as the symptoms worsened. To our knowledge, the clinical utility of 3D-ring CZT SPECT/CT is yet to be demonstrated in patients with lingual Raynaud’s phenomenon.

## 2. Case Presentation

**Figure 1:**
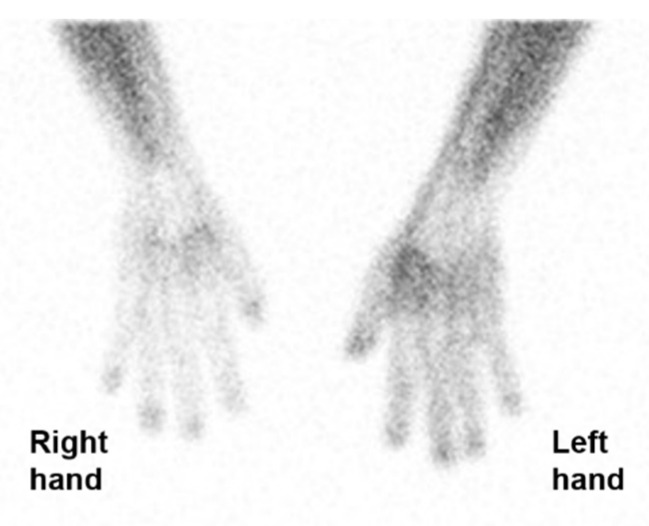
An image of hand perfusion scintigraphy using Tc-99m methylene diphosphonate with chilling stimulation in the right hand. The chilled right hand showed significantly decreased blood pool uptake, showing a right-to-left hand blood pool uptake ratio of 0.47.

A 59-year-old man presented with intermittent episodes of numbness and a tingling sensation in the fingers, toes, and tongue for 1.5 years. These episodes were apparently precipitated by exposure to cold weather or emotional stress but not by the ingestion of cold liquids. During these episodes, he felt discomfort in the tongue, with a mild degree of difficulty in speaking. The patient had no history of rheumatic or arterial occlusive disease and was not taking any medication. Laboratory blood tests revealed that his complete blood count, rheumatoid factor, erythrocyte sedimentation rate, and C-reactive protein level were within normal limits, and antinuclear antibody test results were negative. He underwent perfusion scintigraphy using Tc-99m MDP with cold stimulation of the right hand. For the cold stimulation, the right hand was immersed in cold water for 2 min. After a recovery time of 10 min, a dose of 740 MBq (20 mCi) of Tc-99m MDP was intravenously injected, and the planar blood flow and pool images of the bilateral hands were acquired. The blood pool images of both hands revealed significantly decreased blood pool uptake in the right hand, showing a right-to-left hand blood pool uptake ratio of 0.47 (Figure 1).

**Figure 2:**
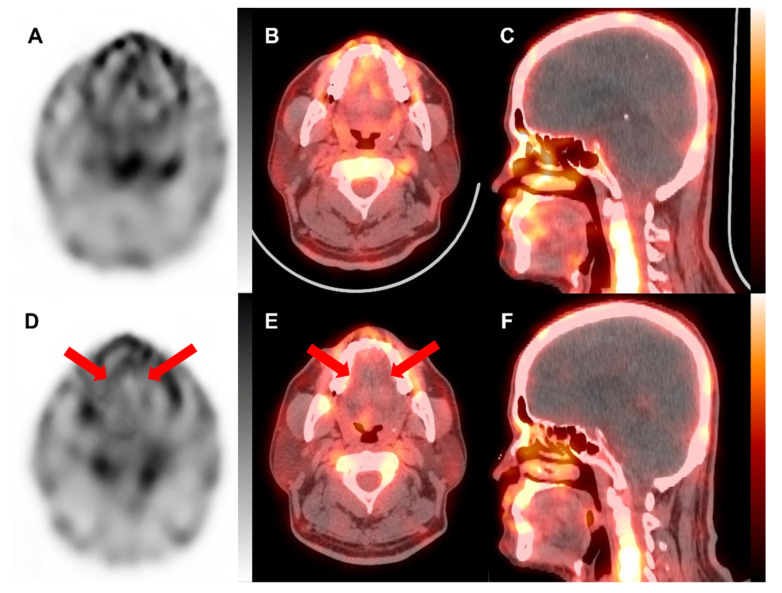
Transaxial single-photon emission computed tomography (SPECT), fused transaxial, and fused sagittal SPECT/computed tomography (CT) images of the tongue on the initial (**A**–**C**) and follow-up (**D**–**F**) blood pool SPECT/CT using Tc-99m methylene diphosphonate. On the initial blood pool SPECT/CT images, heterogeneously increased blood pool uptake was seen in the tongue. On the follow-up SPECT/CT images, blood pool uptake was prominently reduced over the entire area of the tongue (arrows on (**D**,**E**)). In the initial SPECT, the tongue showed unevenly increased intake in various areas, but in the follow-up SPECT, the tongue showed an overall decrease.

The blood pool SPECT/CT images of the tongue were acquired immediately after the acquisition of blood pool images of the bilateral hands (approximately 15 min after the radiotracer injection). The blood pool SPECT/CT of the tongue was performed using a 3D-ring CZT SPECT/CT (Veriton CT, Spectrum Dynamics Medical, Caesarea, Israel) with an imaging acquisition time of 2.5 min. The initial blood pool SPECT/CT of the tongue revealed heterogeneously increased blood pool uptake in the tongue area (Figure 2A–C).

**Figure 3:**
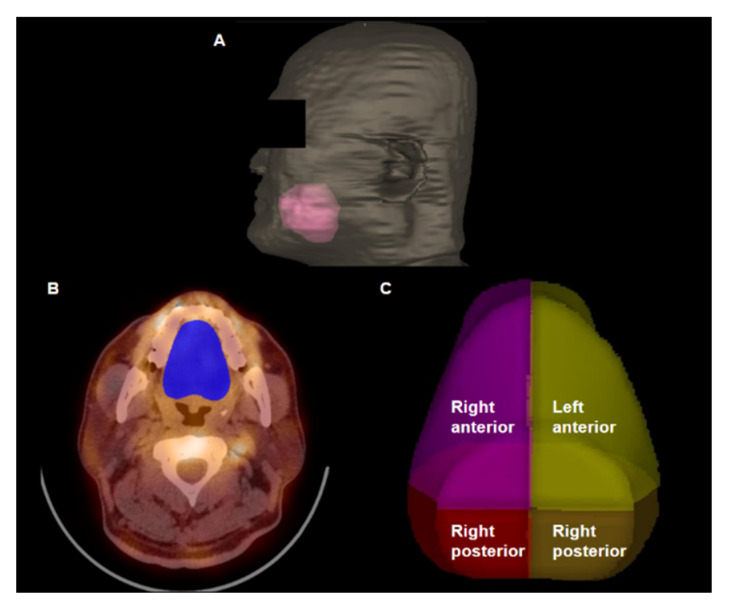
A volume rendering computed tomography image (**A**) and transaxial images (**B**,**C**) of volumes-of-interest of the tongue. The whole tongue area was delineated using a radiation treatment planning software (pink color on (**A**) and blue color on (**B**)). The whole tongue area was divided into four quadrant areas (**C**). Maximum standardized uptake value and total tongue uptake of the whole tongue and four quadrant areas were calculated using these volumes of interest.

Based on the clinical findings and the results of the examinations, the patient was diagnosed with primary Raynaud’s phenomenon and treated with calcium channel blockers and serotonin receptor antagonists. Despite 2 months of medication usage, the patient felt no improvement in symptoms of the fingers, toes, or tongue and discontinued medications by his own choice. Subsequently, the numbness and tingling sensations of the tongue were aggravated, and the patients visited our outpatient clinic again. With suspicion of aggravation of lingual Raynaud’s phenomenon, he underwent a follow-up blood pool SPECT/CT of the tongue. A follow-up blood pool SPECT/CT scan using Tc-99m MDP was performed with the same 3D-ring CZT SPECT/CT, and the SPECT/CT images revealed prominently decreased blood pool uptake in the tongue (Figure 2D–F). For the quantitative analysis of blood pool uptake of the tongue, the whole tongue area was delineated from the surrounding tissue using radiation treatment planning software (Eclipse version 8.9, Varian Medical Systems, Palo Alto, CA, USA), and the tongue area was divided into four quadrant areas (Figure 3). The maximum standardized uptake value (SUV), mean SUV, and total tongue uptake were measured for the entire tongue area and four quadrants of the tongue. SUV was calculated using the following formula:

$$\mathrm{SUV}=\frac{\frac{\mathrm{Measured}\;\mathrm{radioactivity}\;\left(\mathrm{Bq}\right)}{\mathrm{Voxel}\;\mathrm{volume}\;\left(\mathrm{mL}\right)}}{\frac{\mathrm{Decay}\;\mathrm{corrected}\;\mathrm{injected}\;\mathrm{radioactivity}\;\left(\mathrm{Bq}\right)}{\mathrm{Patient}\;\mathrm{weight}\;\left(g\right)}}$$

**Figure 4:**
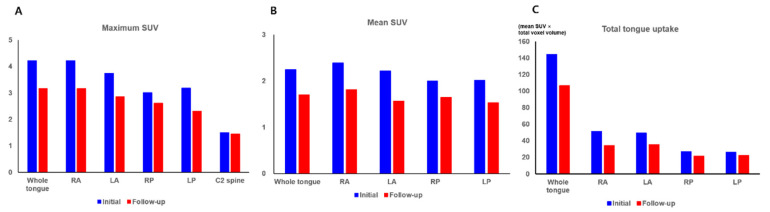
Maximum standardized uptake value (**A**) of the whole tongue, four quadrant areas of the tongue (RA, right anterior; LA, left anterior; RP, right posterior; LP, left posterior) and C2 spine as a reference organ, mean standardized uptake value (**B**) and total tongue uptake (**C**) of the whole tongue and four quadrant areas of the tongue on the initial and follow-up blood pool single-photon emission tomography/computed tomography images.

The maximum SUV was defined as the maximum blood pool uptake in the area. With the threshold set at 40% of the maximum SUV, the voxels within the areas that had a higher SUV than the threshold were delineated, and the mean SUV and total volume of those voxels were measured for each area. The total tongue uptake was calculated by multiplying the mean SUV by the total voxel volume. All four quadrant areas, as well as the whole tongue area, demonstrated a decreased maximum SUV, mean SUV, and total tongue uptake on the follow-up SPECT/CT images as compared with the initial SPECT/CT images (Figure 4; Table 1). Based on the findings of the blood pool SPECT/CT images, lingual Raynaud’s phenomenon was considered to be aggravated, and the patient was instructed to continue the medications as indicated.

## 3. Discussion

The mechanism of Raynaud’s phenomenon remains unknown. In previous studies, increased local vascular hyperreactivity, sympathetic nervous system activity, and various hormonal factors have been suggested as potential mechanisms of Raynaud’s phenomenon [1,10]. Because of sufficient blood supply and collateral circulation, the tongue is considered a rare location of ischemic events; however, several cases of lingual Raynaud’s phenomenon have been reported in both primary and secondary forms [4,11,12,13,14]. Lingual Raynaud’s phenomenon causes various symptoms, such as paresthesia, ulceration, tongue spasm, and dysarthria; however, owing to the intermittent and temporary nature of its symptoms, the disease is often difficult to diagnose and can be overlooked for years [13,15]. The treatment of lingual Raynaud’s phenomenon is similar to that of Raynaud’s phenomenon in the fingers and toes, and successful management has been reported using calcium channel blockers [13,15].

In previous studies that performed perfusion scintigraphy without cold stimulation, patients with Raynaud’s phenomenon showed significantly lower blood flow and pool uptake in the finger areas than healthy participants [6,16]. Furthermore, in another previous study with a double-blind crossover design, uptake of hand areas on perfusion scintigraphy significantly increased after treatment with a serotonin receptor antagonist, whereas it decreased after placebo [17]. The results of these studies suggest that uptake of fingers on perfusion scintigraphy could reflect the perfusion status of fingers and could be used to assess disease severity and treatment response to Raynaud’s phenomenon [5,18]. However, all previous studies were performed with planar gamma scintigraphy, and because of the difficulty of planar gamma imaging of the tongue, no reports have revealed perfusion scintigraphy findings in patients with lingual Raynaud’s phenomenon. In this case, we used Veriton CZT SPECT/CT to image the blood pool uptake in the tongue. This SPECT/CT system combines CZT detectors with a 360° ring-configuration geometry to maximize sensitivity and image quality while reducing the scanning time [7,8,9]. In a previous study, the Veriton CZT SPECT/CT showed superior count sensitivity, higher energy resolution, and better image contrast than conventional SPECT [8]. Our case demonstrated that blood pool SPECT/CT images of the tongue were successfully acquired with the Veriton SPECT/CT scanner with a relatively short scanning time, and we were able to compare blood pool uptake of the tongue using quantitative parameters between initial and follow-up SPECT/CT images. The patients in our case showed decreased blood pool uptake of the tongue on follow-up SPECT/CT, which was performed at the time of worsening lingual symptoms after cessation of the medications. On follow-up blood pool SPECT/CT, the maximum SUV and total tongue uptake decreased by over 25% as compared with the initial SPECT/CT, and the blood pool uptake of the anterior portion of the tongue was prominently decreased. Taking this finding into account, the blood pool uptake of the tongue could be related to the severity of symptoms in lingual Raynaud’s phenomenon. Therefore, blood pool imaging of the tongue using 3D-ring CZT SPECT/CT may have a clinical role in evaluating disease severity and treatment effects in patients with lingual Raynaud’s phenomenon. However, further evaluation is needed to validate the use of SPECT/CT imaging for lingual Raynaud’s phenomenon. Furthermore, to enhance the use of quantitative parameters of SPECT/CT images, the reproducibility of SPECT/CT quantitative parameters and attenuation correction method of SPECT images should also be validated in future studies.

## 4. Conclusions

This clinical case illustrates that the blood pool uptake of the tongue can be acquired using a 3D-ring CZT scanner, and the uptake of the tongue on blood pool SPECT/CT was related to symptoms in patients with lingual Raynaud’s phenomenon. In future studies, blood pool SPECT/CT imaging of the tongue may be used as an imaging modality to assess disease severity and treatment effects in patients with lingual Raynaud’s phenomenon.

## Figures and Tables

**Table 1:** Blood pool uptake and total tongue uptake on the initial and follow-up blood pool tongue single-photon emission tomography/computed tomography images.

Variables		Whole Tongue	RA	LA	RP	LP
Maximum SUV	Initial	4.22	4.22	3.75	3.02	3.18
	Follow-up	3.16	3.16	2.86	2.61	2.31
	Δmaximum SUV	−25.12%	−25.12%	−23.73%	−13.58%	−27.36%
Mean SUV	Initial	2.25	2.39	2.22	2.00	2.02
	Follow-up	1.70	1.82	1.57	1.65	1.53
	Δmean SUV	−24.44%	−23.85%	−29.28%	−17.50%	−24.26%
Total tongue uptake	Initial	144.63	51.46	49.46	27.12	26.62
	Follow-up	106.66	34.43	35.69	21.30	22.57
	Δtotal tongue uptake	−26.25%	−33.08%	−27.85%	−21.45%	−15.23% ^1^

^1^ (Δparameter) = [(parameter on follow-up SPECT/CT) − (parameter on initial SPECT/CT)]/(parameter on initial SPECT/CT) × 100. LA, left anterior; LP, left posterior; RA, right anterior; RP, right posterior; SUV, standardized uptake value; SPECT/CT, single-photon emission computed tomography/computed tomography.

## Data Availability

Not applicable.
